# Glucosinolates are produced in trichomes of *Arabidopsis thaliana*

**DOI:** 10.3389/fpls.2012.00242

**Published:** 2012-10-30

**Authors:** Henning Frerigmann, Christoph Böttcher, Dunja Baatout, Tamara Gigolashvili

**Affiliations:** ^1^Universität zu Köln, Biozentrum Köln, Botanisches InstitutKöln, Germany; ^2^Department of Stress and Developmental Biology, Leibniz Institute of Plant BiochemistryHalle/Saale, Germany

**Keywords:** aliphatic glucosinolates (AGS), *Arabidopsis*, indolic glucosinolates (IGS), MYB-transcription factors, MYB51, MYB28, regulation of glucosinolate biosynthesis, trichomes

## Abstract

Glucosinolates (GS) are important plant secondary metabolites in plant resistance to herbivores, bacteria, and fungi, which have been shown to be accumulating in different organs and tissue types at varying concentrations. There are more than 200 GS species found in order Brassicales and presence of these compounds is well documented on organ-specific but not on cell-specific level. We used UPLC/ESI-QTOF-MS to measure the presence of GS and qRT-PCR to analyse the expression of GS biosynthetic and regulatory genes in isolated *Arabidopsis thaliana* trichomes. Trichomes of *Arabidopsis* are shown to synthesize chemoprotective aliphatic glucosinolates (AGS) and indolic glucosinolates (IGS), which are known for their biological activities against fungi, bacterial pathogens, or herbivores. UPLC/ESI-QTOF-MS analysis of various IGS mutants reveal increased or decreased levels of IGS in trichomes of gain- and loss-of-function mutants correspondingly. Using *pMYB51/HIG1-uidA* and *pMYB28/PMG1/HAG1-uidA* reporter plants we demonstrate that production of these important compounds is activated in trichomes of leaves or inflorescences in response to wounding. Since trichomes represent the first interface in plant-environment interactions, the possible role of GS containing trichomes in plant defense or signaling is discussed.

## Introduction

Due to their sessile life style plants are exposed to numerous stresses, including varying light intensities, wind, extreme temperatures, shortage of water and nutrients, pathogen, and herbivore attack. The plant epidermis and epidermis-derived trichomes represent the first interface in plant-environment interactions. It is therefore not surprising that trichomes often contain defense metabolites (Levin, 1973). Multicellular glandular trichomes, which are found on the leaf surface of many plant species such as *Solanum habrochaites* (wild tomato), *Nicotiana tabacum* (tobacco), *Ocimum basilicum* (sweet basil), and *Mentha piperita* (peppermint) are able to synthesize and/or secrete various chemical compounds such as organic acids, polysaccharides, proteins, terpenes, phenolic compounds, glucose esters, and salts (Kelsey, 1984; Navasero and Ramaswamy, 1991; Walters et al., 1991; Gershenzon et al., 1992; McCaskill et al., 1992; Wang et al., 2001). By contrast, the leaf hairs of *Arabidopsis thaliana* are non-glandular single-cell hairs that develop from epidermal cells and are found on most aerial organs (Hülskamp and Schnittger, 1998). Generally, the non-glandular trichomes are believed to be physically defensive structures, and it has been demonstrated that induced plant response to herbivory correlates with an increased number of trichomes per leaf (Mauricio and Rausher, 1997; Agrawal, 2000; Clauss et al., 2006). It is also reported that the production of trichomes is correlated with reduced damage from insect herbivores in natural populations of the perennial herb *Arabidopsis lyrata* (Kivimaki et al., 2007). Furthermore, the specialist herbivore *Pieris rapae* performed better on trichomeless *gl1* mutant than on wild-type (Col-0) plants (Reymond et al., 2004). Finally, genome-wide gene expression analyses of trichomes (Affymetrix ATH-1 chip) revealed high transcript levels of some genes involved in anthocyanin, flavonoid, and glucosinolate (GS) pathways compared to leaves (Jakoby et al., 2008; Marks et al., 2008). Such biosynthesis of secondary and defense compounds suggest an important role of trichomes in plant defense and protection (Jakoby et al., 2008). Although there are many reports that link glandular and non-glandular trichomes with herbivore attacks (Ehleringer et al., 1976; Wagner, 1991; Simmons and Gurr, 2005), the ecological significance of non-grandular trichomes in chemical defence is still a matter of debate. It is even not clear if corresponding mRNAs (for example, involved in the biosynthesis of secondary compounds) or their end products (corresponding metabolites) are indeed present in the trichomes of the model *Arabidopsis* plant.

Here, we report for the first time that trichomes of *Arabidopsis* are able to synthesize a class of phytoprotective secondary compounds known as GS. There are more than 200 different GS known in order Brassicales (Clarke, 2010) and trichomes of *Arabidopsis* build up the aliphatic glucosinolates (AGS) and indolic glucosinolates (IGS). Trichomes of the loss-of- and gain-of-function mutants of IGS biosynthetic and regulatory genes accumulated different levels of IGS than wild-type trichomes. We demonstrate that not only mRNAs of genes required for the biosynthesis and degradation of GS, but also the mRNAs of MYB transcription factors controlling biosynthesis of these GS are present in trichomes. Expression of MYB transcription factors is rapidly induced upon tissue damage suggesting possible role of GS in plant defense in *Arabidopsis*. We believe that the ability to activate GS biosynthesis in trichomes could be an important feature of plants during plant biotic interactions.

## Materials and methods

### Plant growth

*A. thaliana* (Columbia 0) plants were grown in a growth chamber under an 8 h light (120 μmol m^−2^s^−1^)/16 h dark regime at day/night temperatures of 21°C/18°C and at 40% relative humidity.

### Gain- and loss-of-function *Arabidopsis* mutants

All *Arabidopsis* lines used here are in the Columbia (Col-0) background and were isolated as reported previously. The T-DNA insertion mutant in *MYB51/HIG1* (*High Indolic Glucosinolate 1*) gene is a GABI-Kat line [GK228B12; (Gigolashvili et al., 2007a)] and the gain-of-function mutant *HIG1-1D* of the *MYB51/HIG1* gene was isolated from a population of activation-tagged plants (Gigolashvili et al., 2007a). The *cyp79b2cyp79b3* mutant was previously isolated by (Zhao et al., 2002) and seeds were kindly provided by the authors.

### Promoters of R2R3 MYB transcription factors and histochemical GUS staining

The cloning procedure for the promoters of *MYB51/HIG1* and *MYB28/PMG1/HAG1* was performed as previously described (Gigolashvili et al., 2007a,b).

Induced expression of *ProMYB51/HIG1-uidA* and *ProMY28/PMG1/HAG1-uidA* constructs in trichomes after wounding was analyzed either on entire plants, which were still grown in pots, or on detached leaf and stem pieces. Leaves of *ProMYB51/HIG1-uidA* reporter transgenic lines and inflorescences of *ProMYB28/PMG1/HAG1-uidA* transgenic lines were wounded by scalpel or damaged by small scissors, which was immediately followed by β-glucuronidase staining in a 0.1% X-Gluc solution as reported previously (Gigolashvili et al., 2007a). After overnight incubation at 37°C tissues were destained in 70% ethanol and analyzed microscopically.

### Isolation of trichomes

The preparation method was modified from (Marks et al., 2008). In short, 1.5 g freshly harvested leaf material and 50 mg glass beads (60/80 μm) were mixed and vortexed with 15 ml PBS/EGTA-buffer (139 mM KCl, 10 mM K_2_HPO_4_, 2 mM KH_2_PO_4_, pH 7,4; 50 mM EGTA pH 7,5) in a 50-ml Falcon tube. The procedure (30 s vortexing and 30 s chilling at 4°C) was repeated four times. The supernatant was poured into a new test tube and the leaf material was washed twice with PBS. The supernatants were combined and filtered over a 100 μm sieve. The sieve was rinsed with PBS to collect the trichomes. The solution was centrifuged for 90 s at 150× g.

To confirm the integrity of the isolated trichomes they were examined by light microscopy (A1 A–C) and frozen at −80°C, or freeze-dried for further analysis. To find out if applied isolation procedure resulted in the damage of cell membranes, isolated trichomes were stained by trypan blue followed by several washing steps and microscopical examination of single trichomes (A1D–F).

### RNA extraction and expression analysis

Total RNA was isolated from trichomes by the Trisure method (BIOLINE), according to the manufacturer's instructions. A maximum of 5 μg RNA was reverse-transcribed using SuperScriptII Reverse Transcriptase (Life Technologies). Gene expression was analyzed by real-time quantitative analysis (qRT-PCR) using the fluorescent intercalating dye SYBR-Green in a GeneAmp_5700 Sequence Detection System (Applied Biosystems). To amplify gene targets a standard qRT-PCR protocol from Life Technologies was applied: 95°C for 10 min denaturation, 40 cycles of 95°C for 15 s and 60°C for 1 min annealing and elongation in one step. The *Arabidopsis At2g28390* gene belonging to the SAND family was used as an internal standard (Czechowski et al., 2005). Relative quantification of expression levels was performed using the comparative Ct method, and the calculated relative expression values were normalized to the wild-type expression level (wild type = 1) or to the expression levels of the same gene in leaves (leaves = 1). Primer sequences for the qRT-PCR are listed in A1. Three biological and two technical replicates were used for the analysis.

### Preparation of methanolic trichome and leaf extracts and analysis by UPLC/ESI-QTOF-MS

Freeze-dried leaf/trichome material (7–12 mg) was accurately weighed into 2-mL tubes. After addition of steel balls (Ø2 mm, Retsch) and cooling in liquid nitrogen, the samples were homogenized for 4 min at 30 Hz using a MM301 ball mill (Retsch) and kept frozen on dry ice until extraction. For that purpose, 200 μL 80% aqueous methanol, precooled at −40°C, were added. Afterwards, the samples were immediately vortexed for 20 s, sonicated for 10 min at 20°C and centrifuged for 10 min at 19,000× g. The supernatants were transferred to new 2-ml tubes and the remaining pellets subjected to a second extraction using 200 μL 80% aqueous methanol. The combined extracts were evaporated to dryness in a vacuum centrifuge at 30°C, reconstituted in 10 μL 40% aqueous methanol per mg dry weight, sonicated for 10 min at 20°C and centrifuged for 10 min at 19,000× g. The supernatants (2.8 μL, full loop injection) were separated on an Acquity UPLC system (Waters) equipped with a HSS T3 column (100 × 1.0 mm, particle size 1.8 μm; Waters) using the following gradient program at a flow rate of 150 μL/min: 0–1 min, isocratic 95% A (water, 0.1% formic acid), 5% B (acetonitrile, 0.1% formic acid); 1–16 min, linear from 5 to 95% B; 16–18 min, isocratic 95% B; 18–20 min, isocratic 5% B. Eluted compounds were detected from *m/z* 100–1000 using a MicroTOF-Q hybrid quadrupole time-of-flight mass spectrometer (Bruker Daltonics) equipped with an Apollo II electrospray ion source in positive and negative ion mode. For instrument settings and calibration see (Bottcher et al., 2009). Target compounds were relatively quantified by integrating the following extracted ion chromatograms (*m/z*-width ±0.02): 8-MeSO-octyl-GS (C_16_H_31_NO_10_S_3_), *t*_r_= 192 s, *m/z* 492.10 [M-H]^−^; 7-MeSO-heptyl-GS (C_15_H_29_NO_10_S_3_), *t*_r_ = 143 s, *m/z* 478.09 [M-H]^−^; 4-MeSO-butyl-GS (C_12_H_23_NO_10_S_3_), *t*_r_ = 42 s, *m/z* 436.04 [M-H]^−^; I3M-GS (C_16_H_20_N_2_O_9_S_2_), *t*_r_ = 168 s, *m/z* 447.05 [M-H]^−^; 4-MeO-I3M-GS (C_17_H_22_N_2_O_10_S_2_), *t*_r_ = 215 s, 477.06 [M-H]^−^; 1-MeO-I3M-GS (C_17_H_22_N_2_O_10_S_2_), *t*_r_ = 252 s, 477.06 [M-H]^−^; 8-MeSO-octyl-NCS (C_10_H_19_NOS_2_), *t*_r_ = 492 s, *m/z* 256.08 [M+Na]^+^; 7-MeSO-heptyl-NCS (C_9_H_17_NOS_2_), *t*_r_ = 441 s, *m/z* 242.06 [M+Na]^+^; 4-MeSO-butyl-NCS (C_6_H_11_NOS_2_), *t*_r_ = 270 s, *m/z* 200.02 [M+Na]^+^; 8-MeSO-octyl-CN (C_10_H_19_NOS), *t*_r_ = 334 s, *m/z* 224.11 [M+Na]^+^; 7-MeSO-heptyl-CN (C_9_H_17_NOS), *t*_r_ = 275 s, *m/z* 210.09 [M+Na]^+^; 4-MeSO-butyl-NCS (C_6_H_11_NOS), *t*_r_ = 68 s, *m/z* 168.05 [M+Na]^+^; 8-MeSO-octyl-NH_2_ (C_9_H_21_NOS), *t*_r_ = 144 s, *m/z* 192.14 [M+H]^+^. Other known AGS in *Arabidopsis* were under detection limits in methanolic extracts of trichomes. GS detected in trichome extracts were referenced and quantified using HPLC of *Arabidopsis* leaf extracts as described previously (Gigolashvili et al., 2007b). Isothiocyanates (ITCs) were validated using commercially available standards (LKT Laboratories). Nitriles were putatively identified using accurate mass measurements and tandem-MS experiments. Accurate mass measurements and collision-induced mass spectra are listed in A2 and A3. 8-MeSO-octyl-NH_2_ was prepared from the corresponding ITC by acidic hydrolysis.

## Results

### Glucosinolate biosynthetic genes are expressed in trichomes

**Figure 1:**
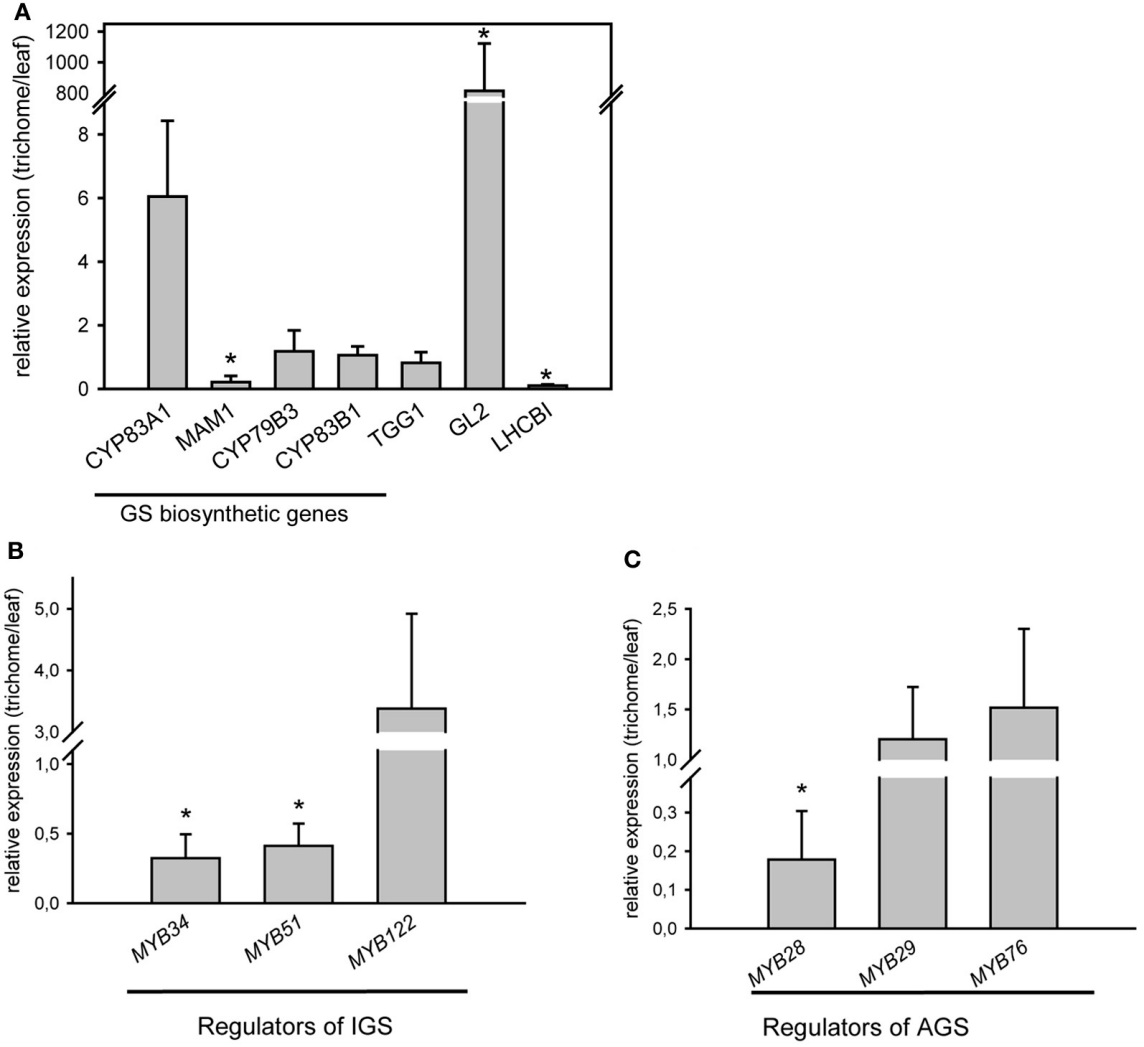
**Relative expression levels of GS biosynthetic and regulatory genes in trichomes of wild-type plants (Col-0). (A)** Relative expression level of *CYP79B3, MAM1, CYP83B1, CYP83A1*, *TGG1, GL2*, and *LHCBI* in trichomes of wild-type plants; **(B)** Relative expression of indolic glucosinolate (IGS) regulators: *MYB51/HIG1, MYB34* and *MYB122;* **(C)** Relative expression of aliphatic glucosinolate (AGS) regulators: *MYB28, MYB29* and *MYB76*. Expression analysis of genes required for GS biosynthesis was performed on detached trichomes, which were collected as described in the Materials and Methods. *GLABRA2* gene *(GL2)*, which is mainly found in trichomes and *LHCBI*, which is expected to be found in photosynthetically active leaf tissue have been used as positive and negative controls for corresponding tissue types. Three biological and two technical replicates were used for the analysis. Error bars show SE of the mean. Relative gene expression levels are shown in trichomes vs. leaves (leaves = 1). Significantly different values in trichomes vs. leaves are marked with asterisk (Student's *t*-test, *P* < 0.05).

qRT-PCR analysis for the expression of GS biosynthetic genes and regulators in wild type *Arabidopsis* trichomes was performed. Transcriptional profiling of representative genes involved in GS metabolism demonstrated the presence of transcripts belonging to the GS pathway. Figure 1A depicts expression levels of exemplarily chosen genes required for the biosynthesis of aliphatic (*MAM1, CYP83A1*), indolic (*CYP79B3, CYP83B1*) GS (corresponding biosynthetic pathways are shown in A2), or breakdown of GS (*TGG1*) in isolated trichome extracts compared to expression levels in whole leaves. These results confirm the presence of biosynthetic and breakdown genes of the GS-myrosinase system in trichomes.

However, not only GS biosynthetic genes, but also genes encoding transcription factors, that are known to control the biosynthesis of GS (Gigolashvili et al., 2007a,b, 2008; Hirai et al., 2007; Sonderby et al., 2007, 2010; Malitsky et al., 2008) are transcribed in trichomes. As illustrated in Figures 1B,C, mRNA of both groups of transcription factors, namely *MYB28 (PMG1/HAG1), MYB29 (PMG2/HAG2)*, and *MYB76 (HAG3)* as well as *MYB51/HIG1, MYB122 (HIG2)*, and *MYB34 (ATR1)*, which regulate biosynthesis of AGS and IGS (A2), respectively, are present in *Arabidopsis* trichomes.

### Indolic and aliphatic glucosinolates are present in trichome cells

**Figure 2:**
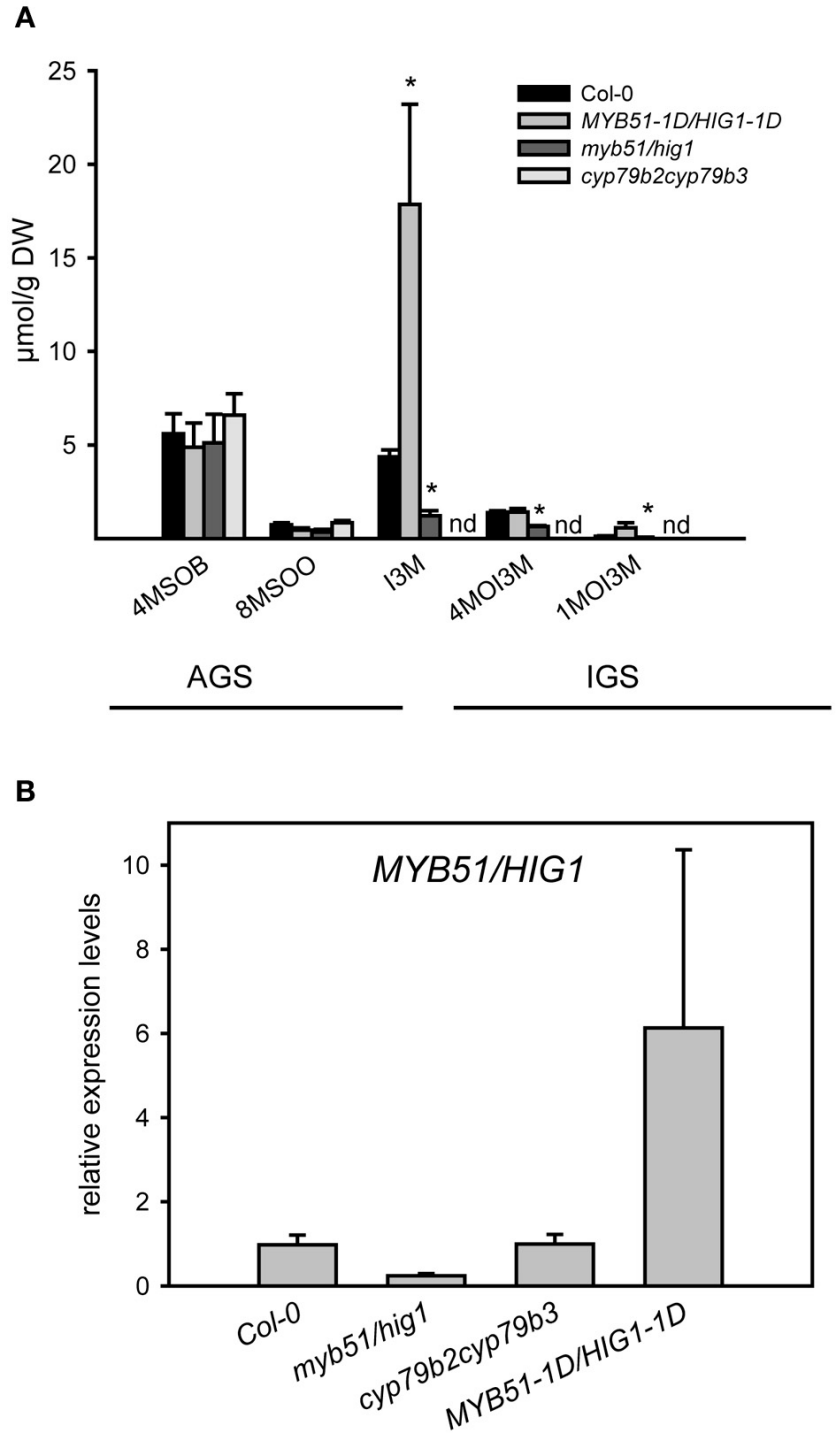
**Glucosinolate production in trichomes of *myb51/hig1, MYB51-1D/HIG1-1D* and *cyp79b2cyp79b3* mutants. (A)** Aliphatic glucosinolates (AGS) and indolic (IGS) glucosinolates have been quantified in methanolic extracts of detached trichomes of *MYB51-1D/HIG1-1D, myb51/hig1, cyp79b2cyp79b3*. 4-MeSO-butyl-GS (4MSOB), 8-MeSO-octyl-GS (8MSOO), I3M-GS, 4-MeO-I3M-GS (4MOI3M) and 1-MeO-I3M-GS (1MOI3M) are shown. **(B)** Relative steady-state level of *MYB51/HIG1* mRNA in trichomes of GS mutants and wild-type (Col-0) plants. Comparative expression analysis of *MYB51*/*HIG1* in *myb51/hig1, MYB51-1D/HIG1-1D* and *cyp79b2cyp79b3* mutants vs. Col-0 (=1) was performed on detached trichomes. Trichomes were collected as described in “Materials and Methods.” Three biological and two technical replicates were used for the qRT-PCR analysis. Error bars show SE of the mean. nd- not detected. Significantly different values from the wild type at *P* < 0.05 (Student's *t*-test) are marked by asterisk.

The presence of transcripts of structural and regulatory genes of GS biosynthesis in trichomes suggests that these chemoprotective secondary metabolites are synthesized in these cells. To further substantiate this finding, we screened for AGS and IGS in extracts of isolated trichomes of wild-type plants and IGS mutants, using UPLC/ESI-QTOF-MS as described previously (Bottcher et al., 2008). As shown in Figure 2A, the trichomes of wild-type plants and mutants accumulated some methylsulphinylalkyl (4-MeSO-butyl-, 8-MeSO-octyl-GS) and indolic (I3M-, 4-MeO-I3M- and 1-MeO-I3M-GS) GS, whereas methylthioalkyl GS were below the detection limit. Furthermore, analyses of GS in trichomes of loss-of-function IGS mutants revealed lower IGS levels in trichomes of *myb51/hig1-1* and *cyp79b2cyp79b3* mutant plants than in wild type (Col-0). Observed GS levels in trichomes of Col-0, *myb51/hig1-1* and *MYB51-1D/HIG1-1D* correlated well with the amount of *MYB51/HIG1* mRNA (Figure 2B). The *cyp79b2cyp79b3* mutant, which is defective in the production of IAOx-derived metabolites (Zhao et al., 2002), did not contain any detectable amounts of I3M-, 4-MeO-I3M- or 1-MeO-I3M-GS (Figure 2A). Conversely, the gain-of-function mutant *MYB51-1D/HIG1-1D* contained higher than wild-type levels of I3M- and 4-MeO-I3M-GS. Amounts of all measured GS in trichomes of wild type and mutant plants were present at about 100 times lower levels than in leaves (A3). However, even these amount of GS have, most probably, an important biological activity *in vivo*, as, for example, comparable amounts of GS have been reported to have sufficient antimicrobial activity *in vitro* (Tierens et al., 2001).

### Promoters of R2R3 MYBs controlling biosynthesis of glucosinolates are induced upon physical damage in trichomes

**Figure 3:**
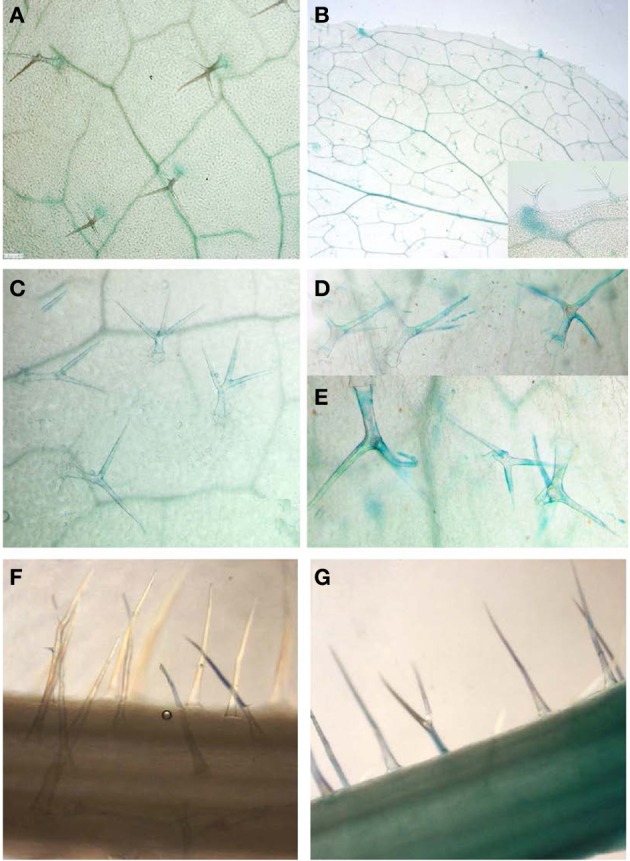
**Activity of *MYB51/HIG1* and *MYB28/PMG1/HAG1* promoters controlling the biosynthesis of IGS and AGS in *Arabidopsis.* (A,B)** Basal activities of *ProMYB51/HIG1-uidA* **(A)** and *ProMYB28/PMG1/HAG1-uidA* **(B)** in trichomes of detached *Arabidopsis* rosette leaves. **(C,D)** Expression of *ProMYB51/HIG1-uidA* construct in trichomes of entire plants with roots before (**C**) and after **(D)** wounding of rosette leaves. **(E)** Induced expression of the *ProMYB51/HIG1-uidA* construct in mechanically damaged trichomes of entire plants with roots. Damaged trichomes are still attached to the surface of leaves. **(F,G)** Induced expression of *ProMY28/PMG1/HAG1-uidA* construct in trichomes before (**F**) and after **(G)** wounding of inflorescences. Leaves and inflorescences of *ProMYB51/HIG1-uidA* and *ProMY28/PMG1/HAG1-uidA* reporter transgenic lines were wounded by scalpel (or scissors) making five to six repeating 0,5 cm cuts along the corresponding tissue, and were immediately used for β-glucuronidase staining. Wounding response was restricted to the inflicted leaf and no systemic response in the trichomes of other leaves was observed.

Based on the finding that GS are synthesized in trichomes (Figure 2A) along with the presence of known MYB regulators (Figures 1B,C), we re-analyzed the activity of promoter-reporter constructs of the main indolic (*MYB51/HIG1*) and aliphatic (*MYB28/PMG1/HAG1*) GS regulators. Indeed, as previously reported (Gigolashvili et al., 2007a,b), we could confirm activity of *MYB51/HIG1* and *MYB28/PMG1/HAG1* promoters in leaf hairs of *Arabidopsis* rosette leaves and hairs of inflorescences correspondingly (Figures 3A,B).

To identify whether the biosynthesis of GS in trichomes is also regulated by wounding, leaves and trichomes of *ProMYB51/HIG1-uidA* reporter transgenic lines and inflorescences of *ProMYB28/PMG1/HAG1-uidA* transgenic lines were wounded by scalpel or damaged by small scissors, which was immediately followed by β-glucuronidase staining and microscopic analysis of trichomes. To avoid additional induction of reporter constructs by detachment of leaves in experiment presented on Figures 3C–E, wounding and GUS staining were performed on entire plants with roots. 3C–E demonstrate the induction of β-glucuronidase activity in both broken trichomes (Figure 3E) and trichomes of wounded leaves (Figure 3D) compared to unwounded ones (Figure 3C) of *ProMYB51/HIG1-uidA* reporter transgenic lines. As for the reporter gene activity in *ProMYB28/PMG1/HAG1-uidA* transgenic lines, it was detectable in trichomes of wounded inflorescences (Figure 3G) against unwounded ones (Figure 3F), but not in leaves (not shown).

**Figure 4:**
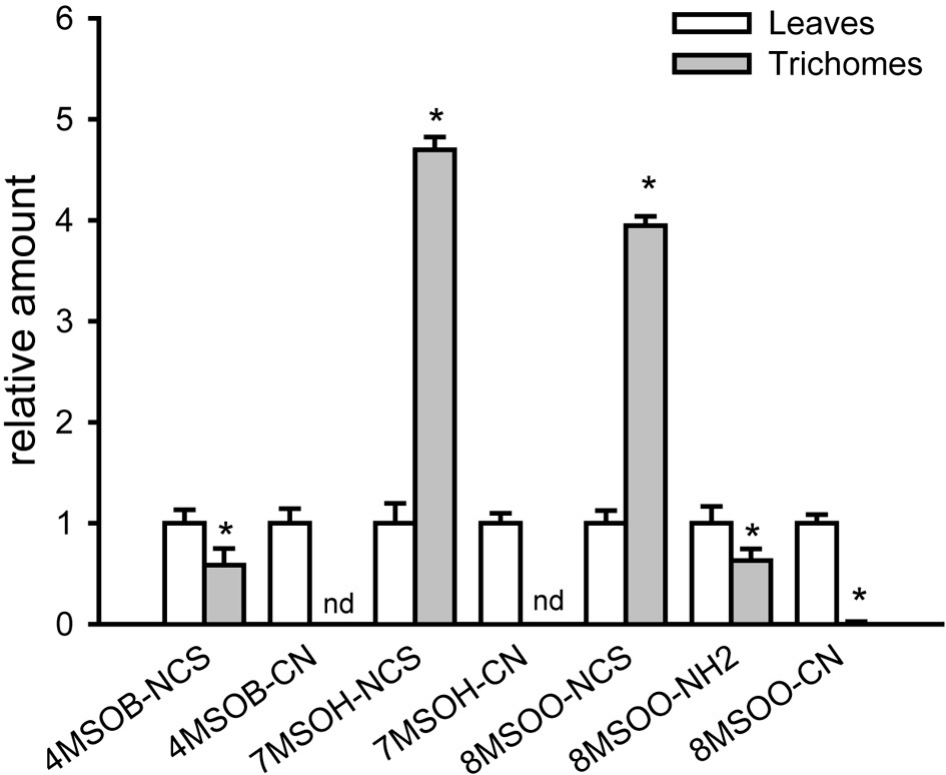
**GS degradation products are present in trichome extracts of wild-type (Col-0) plants.** Relative amounts of GS degradation products in trichomes vs. leaves (peak area in leaves = 1) are shown. Isothiocyanates (4-MeSO-butyl-NCS, 7-MeSO-heptyl-NCS, 8-MeSO-octyl-NCS), nitriles (4-MeSO- butyl-CN, 7-MeSO-heptyl-CN, 8-MeSO-octyl-CN) and 8-MeSO-octyl -derived amine (8-MeSO-octyl-NH2) have been measured in methanolic leaf and trichome extracts using UPLC/ESI-QTOF-MS. Data are normalized to corresponding dry weights of trichomes and leaves. nd- not detected. Significantly different values from the wild type at *P* < 0.05 (Student's *t*-test) are marked by asterisk.

To assess whether the induction of GS biosynthesis correlates with the accumulation of ITCs, which are toxic for other organisms, methanolic extracts of detached trichomes were analyzed for various GS degradation products. The UPLC/ESI-QTOF-MS analyses of trichomes revealed comparable (for 4-MeSO-butyl-NCS) or even higher than in levels (for the 7-MeSO-heptyl-NCS and 8-MeSO-octyl-NCS) levels of ITCs derived from AGS. In addition, we could measure in the trichomes 8-MeSO-octyl-derived amine in amounts comparable with the leaves and traces of 8-MeSO-octyl-derived nitrile (Figure 4). Although we were able to detect nitriles derived from 4-MeSO-butyl- and 7-MeSO-heptyl-GS in leaves, these compounds were below the detection limit in trichome extracts. Moreover, the degradation products of IGS (e.g., ascorbigens or indole-3-carbaldehydes) were also detectable only in leaf extracts (data not shown). GS degradation products measured in trichomes of Col-0 could be generated during GS degradation or turnover in living cells (*in vivo*) or during isolation of trichomes, which is a mechanical stress *per se*, comparable to a mechanical stress caused by herbivores. Indeed, about 2–5% of trichomes showed a penetration of trypan blue dye into the cytosol (A1D–F), which is an indication of, at least, plasma membrane damage. Taken together, these data indicate that trichomes are able to synthesize GS and to induce their production upon physical stress by inducing MYB transcription factors controlling biosynthesis of AGS and IGS.

## Discussion

The plant epidermis, covered with trichomes is an immediate interface in plant-environment interactions and an active role of trichomes in plant biotic and abiotic interactions has been suggested in numerous ecological studies. For instance, the negative correlation between trichome density and insect feeding and oviposition responses and/or the nutrition of larvae has been shown for *Arabidopsis* and other plant species (Mauricio and Rausher, 1997; Agrawal, 2000). The production of hooked trichomes and thorns has been reported for the herb plant *Mentzelia pumila*, which seems to be a direct plant defense mechanism against herbivore attack (Eisner et al., 1998). The toxic content of non-glandular trichomes of *Glycine max* (Levin, 1973) has been described to immobilize pathogens after cell disruption. Glandular trichomes can serve additionally in plant chemical defense, since their glands can exude terpenes, phenolics, alkaloids, or other substances, which are gustatory repellents of herbivores. Finally, microarray analysis revealed the presence of several secondary metabolites genes, including some GS biosynthetic genes, in isolated trichomes, indicating the possible protective function of trichomes (Jakoby et al., 2008; Marks et al., 2008).

### Uncovering glucosinolates in trichomes of *Arabidosis*

In this paper we present data showing the presence of transcripts of GS biosynthesis and breakdown genes, as well as the presence of AGS and IGS in trichomes of *Arabidopsis* by performing qRT-PCR and UPLC/ESI-QTOF-MS analyses of isolated trichomes. Herewith we could provide for the first time evidence for the presence of biologically active secondary compounds, like GS, in non-glandular trichomes of *Arabidopsis*, and, suggest in this way an active role of these compounds of plant hairs in plant-biotic interactions.

Using UPLC/ESI-QTOF-MS-based analysis of isolated trichomes, we show that several IGS and AGS, as well as several aliphatic ITCs, 8-MeSO-octyl-derived amine and minor amounts of 8-MeSO-octyl-derived nitrile are present in trichome extracts of *Arabidopsis* wild-type plants (Figures 2A and 4). Furthermore, using qRT-PCR analysis of isolated trichomes we demonstrated that along with the trichome-specific *GLABRA2 (GL2)* gene, the mRNAs of genes involved in the biosynthesis and regulation of IGS and AGS are also present in trichomes (Figure 1). An expression of leaf-specific gene *LHCBI* was negligible in the cDNA prepared from trichomes, which excluded a possibility of contamination by leaves in our trichome extracts. Our data is in agreement with *in silico* data of (Jakoby et al., 2008) that demonstrated mRNAs of several GS biosynthetic genes, such as *CYP83B1, UGT74B1, SOT16*, or *CYP83A1, UGT74B1, SOT18* in isolated trichomes.

To obtain insight into the constitutive production of GS in trichomes of *Arabidopsis*, we analyzed the accumulation of IGS in a set of IGS mutants. Trichomes of *cyp79b2cyp79b3* and *myb51/hig1* knock-out plants, which are known to be impaired in the production of IGS in leaves also showed decreased IGS levels in trichomes (Figure 2). The *cyp79b2cyp79b3* mutant, which is known to completely lack IGS in leaves, was also not able to accumulate these compounds in trichomes. As expected, the levels of AGS, however, remained unaltered in these mutants. On the contrary, the *MYB51-1D/HIG1-1D* mutant, which is known to accumulate high levels of IGS in leaves (Gigolashvili et al., 2007a), also contained high IGS levels in trichomes. Altogether, these findings demonstrate that GS are synthesized in wild-type trichomes, and their biosynthesis in trichomes is controlled by the same set of biosynthetic and regulatory genes as in the leaves or seeds.

Although the presence of GS in trichomes is presented for the first time in this work, the spatial variations of some GS within the leaves (Shroff et al., 2008) as well as the knowledge about specific S-cells in plants, accumulating higher levels of GS (Koroleva et al., 2000, 2010) has been known since several years. GS accumulating S cells are known to contain about 19 times greater concentration of sulphur (and hence GS) than the surrounding tissues, providing the advantages of protecting the nutrient-rich phloem from herbivores (Shroff et al., 2008; Koroleva et al., 2010). In addition to the S-cells, it has been found that the cells of the outer margin of the leaf accumulate high amounts of sulphur (Koroleva et al., 2010), corresponding well to a specific localization of IGS (Shroff et al., 2008) and providing better defense from the herbivores, which usually bite leaf margins. Another study demonstrated accumulation of GS in the S-like specific root cell layers of canola. The highest GS concentrations were reported to be accumulating in two cell layers just below the outermost periderm layer, with up to 100-fold higher concentrations than the mean value in the root (McCully et al., 2008). GS or GS-degradation products present is these specific root cells may be released into the rhizosphere to act as biologically active compounds during interaction with organisms living in soil.

### How much glucosinolates are produced or needed in trichomes?

In comparison with leaves, levels of GS in trichomes are about 100 times lower, however, even these amounts could be enough to have, for example, an anti-microbial effect. Assuming all 4-MeSO-butyl-GS present in trichomes is converted into 4-MeSO-butyl-NCS, the absolute amounts of 4-MeSO-butyl-NCS generated in trichomes will be comparable with the amounts of 4-MeSO-butyl-NCS reported by Tierens et al., 2001 to have sufficient antibacterial activities *in vitro*. However, the relative amounts of ITCs measured in trichomes were not 100 times lower than in leaves, but for some metabolites (like 4-MeSO-butyl-NCS) were well comparable or for other (like 7-MeSO-heptyl-NCS and 8-MeSO-octyl-NCS) even higher than in leaves. Thus, although trichomes accumulate much lower level of native GS than the leaves, GS breakdown products like ITCs are accumulating at comparable or even higher levels than in leaves, indicating better performance of ITC generation machinery of non-glandurar trichomes of *Arabidopsis* plants. Altogether our study is in agreement with previous works showing only weak correlation between mRNA level and its products like protein levels, enzyme activities or metabolite levels (Gygi et al., 1999; Piques et al., 2009; Sonderby et al., 2010). For some genes, steady-state levels of certain proteins were recorded with respective mRNA transcript levels that varied by as much as 30-fold, at the same time, while the mRNA levels were of the same value, the protein levels varied by more than 20-fold (Gygi et al., 1999). Similar observations demonstrating an uncoupling of GS levels from the level of transcript of GS biosynthetic genes were made during analysis of the regulation of GS biosynthetic pathway (Sonderby et al., 2010). These authors suggested that “this uncoupling of chemotypes from biosynthetic transcripts suggests revising our view of the regulation of GS metabolism from a simple linear transcription factor-promoter model to a more modular system in which transcription factors cause similar chemotypes via non-overlapping regulatory patterns” (Sonderby et al., 2010).

### Production of glucosinolate regulators in trichomes is increased upon wounding

As it was shown previously, the expression of the six *R2R3-MYB* transcription factors in leaves is positively regulated by different environmental stimuli, such as wounding or hormone treatment (Gigolashvili et al., 2007a, 2008; Hirai et al., 2007). To analyze if the production of GS regulators is also activated in trichomes, plants containing a promoter-*uidA* construct of the main indolic (*MYB51/HIG1*) and main aliphatic (*MYB28/PMG1/HAG1*) GS regulators were subjected to mechanical stress followed by β-glucuronidase activity assay. It could indeed be demonstrated that the expression of the main regulator of IGS and AGS in trichomes is induced upon wounding (Figures 3D–G) and this induction was mainly detectable in the trichomes of rosette leaves in the case of *ProMYB51/HIG1-uidA* plants and in the trichomes of inflorescences in *ProMYB28/PMG1/HAG1-uidA* reporter plants. This finding fits well with previous observation demonstrating that leaves and inflorescences are the main sites of IGS and AGS accumulation correspondingly (Brown et al., 2003; Sarsby et al., 2012). Altogether we could show that trichomes of *Arabidopsis* are not only able to synthesize different GS in trichomes of leaves and inflorescences, but are also capable of activating their production upon mechanical stress. However, it remains to be elucidated how different types of GS at different concentrations and in different cell types are used during interactions with various organisms.

### Trichome-localized glucosinolates and their potential in plant-pathogen interactions

Following our discovery of GS biosynthesis pathway in trichomes, the relation of GS biosynthesis in trichomes with the production of biologically active GS catabolism products, which could affect the plant interaction with other organisms were analyzed. Upon disruption of tonoplast membrane of leaf mesophyll cells, vacuolar-localized GS (Agee et al., 2010; Klie et al., 2011; Krueger et al., 2011) are known to be released into the cytosol causing an activation of GS-mirosinase system and production of ESP- and NSP-dependent and independent GS degradation products (Burow et al., 2006, 2009; Wittstock and Burow, 2007; De Vos et al., 2008; Mumm et al., 2008; Burow and Wittstock, 2009). Trichomes of *Arabidopsis* (Col-0) wild-type plants were shown to accumulate diverse aliphatic ITCs, like 4-MeSO-butyl-NCS, 7-MeSO-heptyl-NCS, and 8-MeSO-octyl-NCS, low amounts of 8-MeSO-octyl-derived nitrile (8-MeSO-octyl-CN) and comparable with the level of 4-MeSOB- derived ITC amine (8-MeSO-octyl-NH2), underlying the potential of GS-myrosinase system in this cell type (Figure 4). Higher level of ITC and low levels of nitriles are also in accordance with the presence of myrosinase (*TGG1*) transcripts in trichomes (Figure 1A), and with lower abundance of NSP transcripts in comparison with TGG1 (Marks et al., 2008). Ability to generate ITCs in trichomes could be considered as an advantageous feature of plants as positive role of GS in direct defense is usually attributed to the ITCs, which are toxic upon ingestion and/or contact (Wittstock et al., 2003). A recent study of (Fan et al., 2012), opened new insights about the role of ITCs in plant microbe-interactions and demonstrated that aliphatic ITCs are crucial for robust defenses that underpin non-host resistance in the *Arabidopsis*-*Pseudomonas* pathosystem. It can be, therefore, suggested that ITCs, which can be induced in trichomes, in analogy to ITCs induced in other parts of plants could be required in plant-bacterial interactions and/or plant defense against bacterial pathogens.

Interestingly, among GS catabolism products we detect significant amounts of amine derived from 8-MeSO-octyl-GS. GS-derived amines are known to be generated in living cells in myrosinase-independent (or alternative GS catabolism) pathway as side products of GS degradation. Examples of such pathways are PEN2 and PYK10-mediated metabolism of GSs (Bednarek et al., 2009; Wittstock and Burow, 2010). However, biochemical structures of active molecules generated in PEN2- and PYK10-mediated GS degradation are not known, most probably because they are highly active and are quickly detoxified in the cell. It is only known that IGS, like 4MOI3M and 1MOI3M, but also partially I3M are used as substrates by both PEN2 and PYK10 (Bednarek et al., 2009; Wittstock and Burow, 2010). The facts that some amines could be detected in trichomes of *Arabidopsis* indicate that alternative GS degradation pathway could exist in living trichomes as well. This suggestion is supported by the observation in which we could measure comparably higher levels of IGS in trichomes but were not able to detect the typical degradation products of IGS such as ascorbigen or carbonyles. Thus, it may be that the fate of IGS in trichomes is additionally determined by PEN2 and PYK10-like enzymes. The absence of IGS degradation products in trichome extracts, along with the hardly detectable expression level of *LHCBI* gene, provides additionally an independent proof of purity of trichome extracts. If contaminated by leaves, the metabolite profile of trichomes would phenocopy the profile of leaves, which is obviously not the case.

In principle and depending on the type of pathogen coming into contact with trichomes, all possible GS degradation products could be generated and employed during plant-pathogen interactions. Some of these compounds like ITCs accumulate at higher concentration in trichomes, and therefore could be used in direct defense. Others, like nitriles are present at lower concentrations, and therefore can play a role as signaling molecules. For example, a volatile GS degradation products released from the damaged tissue have been shown to have important function in tri-trophic interactions even at lower concentrations (Wittstock et al., 2003; Burow et al., 2006, 2009; De Vos et al., 2008; Mumm et al., 2008; Burow and Wittstock, 2009).

### Perspectives for glucosinolate research in trichomes

Our data demonstrate that trichomes are not only synthesizing GS at basal levels or regulate their production upon physical stress by inducing MYB transcription factors, but that they are also able to activate the GS breakdown pathway and to accumulate biologically active GS degradation products, like ITC at the levels comparable with leaves. The ability to rapidly release GS degradation compounds from damaged trichomes immediately after contact of plant trichomes with interacting organism, will, most probably, change an interaction scenario and serve for the improvement of plant fitness in complex environments.

Along with GS one may expect the presence of other defensive components in plant trichomes, which could be important in plant-environment interactions. For example, Wienkoop et al. (2004) have reported an accumulation of disease-related proteins like RPP5 like protein, or TIR-NBS-LRR in proteomics study of trichomes of *Arabidopsis*. Alternatively and using the metabolomics approach of primary metabolites in trichomes of *Arabidopsis*, Ebert et al. (2010) reported the presence of various precursors of secondary compounds in plants, like amino acids, fatty acids, lipids, sugars, polyols and indole-3-acetonitrile. Increased accumulation of defensive compounds like protease inhibitors, the emission of volatiles attracting predators and parasites of herbivores (Takabayashi and Dicke, 1996; Geervliet et al., 1997; De Moraes et al., 1998; De Leo et al., 2001) or the reduction in plant nutritional quality for herbivores (Bi et al., 1997) have been reported to affect plant performance and their interaction with enemies. Therefore, the production of any defensive or signaling compounds in plants will be expected to result in changed plant environmental interaction, reduced performance of “plant enemies,” better performance of “plant associates,” thereby promoting plant survival in a continuously changing environment.

It can be assumed that in natural environments, where plants are constantly interacting with other organisms or are under the attack by a wide range of plant enemies, trichome-mediated chemical interactions may have a pronounced ecological importance for plants.

### Conflict of interest statement

The authors declare that the research was conducted in the absence of any commercial or financial relationships that could be construed as a potential conflict of interest.

## Figures and Tables

**Figure A1:**
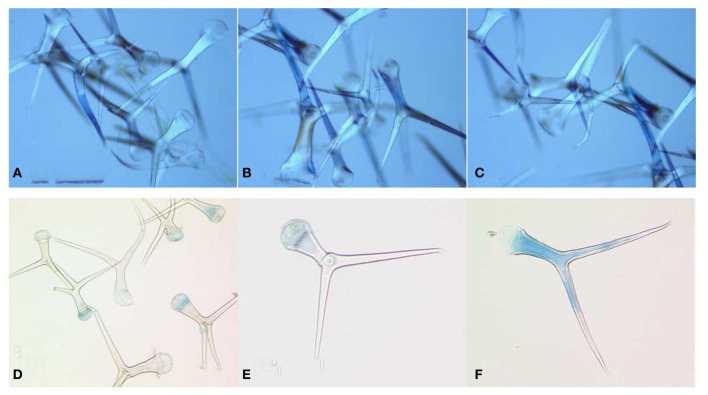
**Microscopic image of isolated trichomes. (A–C)** show three different biological replicates of independent trichome isolations. **(D–F)** Analysis of trichom intactness after trypan blue staining; **(D**) Pool of isolated trichomes after trypan blue staining; **(E)** Intact trichome. Trypan blue is only visible on the base of trichome where it was attached to the epidermal cells; **(F)** shows damaged trichome. Cytoplasm of cell is stained in blue because of destruction of inner membranes and dye penetration into the cell. About 2–5% of trichomes showed visible staining of the cell cytoplasm.

**Figure A2:**
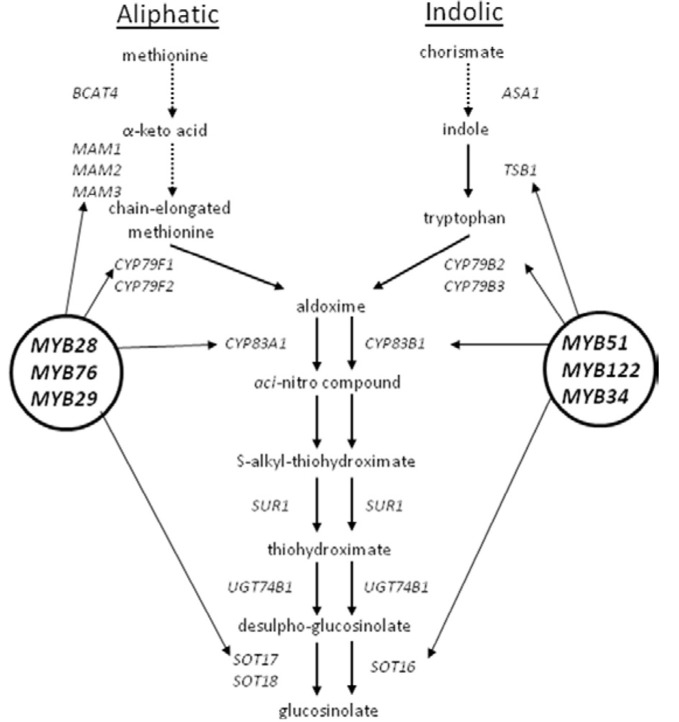
**Pathways for the biosynthesis of aliphatic (left) and indolic (right) glucosinolates**.

**Figure A3:**
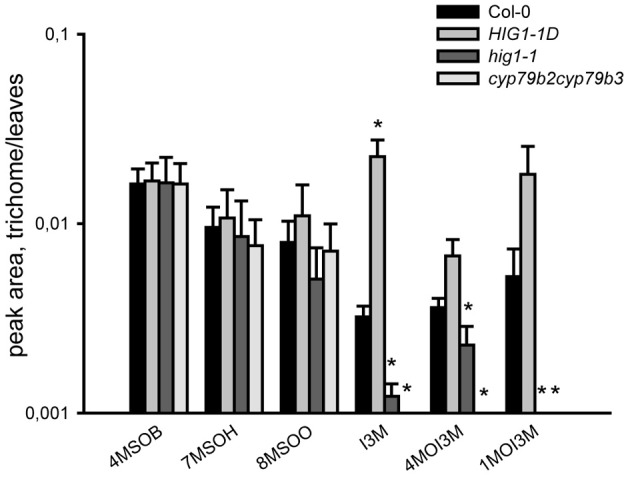
**Relative quantification of GS in methanolic extracts of detached trichomes vs. leaves of Col-0, *MYB51-1D/HIG1-1D, myb51/hig1, cyp79b2cyp79b3* using UPLC/ESI-QTOF-MS.** GS levels in trichomes are shown in relation to GS levels in leaves. Trichomes were collected as described in “Materials and Methods.” Three biological and two technical replicates were used for the analysis. Error bars show SE of the mean. Peak areas are normalized to dry weights of trichomes and leaves. Significantly different values from the wild type at *P* < 0.05 (Student's *t*-test) are marked by asterisk.

## Appendix

**Table A1:** **Primer sequences for the Real-time PCR analysis**.

MYB28_Fw	At5g61420	TCCCTGACAAATACTCTTGCTGAAT	MYB28
MYB28_Rv		CATTGTGGTTATCTCCTCCGAATT	
MYB29_Fw	At5g07690	GAAATATGATGCTTCCTTGAGCTCC	MYB29
MYB29_RV		ACGGTGTAGAGCTGATCAAGGTTC	
MYB76_Fw	At5g07700	ACGTTTTGGACGATCGAGCTCTAC	MYB76
MYB76_RV		TGATTGAGAGAACGAGTCTGGGAGT	
MYB34/ATR1_Fw	AT5G60890	CACGACTGTCGATAATTTTGGGTT	MYB34
MYB34/ATR1_Rv		CATATTGTCATCTTCGTTCCAGGA	
MYB51_Fw	AT1G18570	CTACAAGTGTTTCCGTTGACTCTGAA	MYB51
MYB51_Rv		ACGAAATTATCGCAGTACATTAGAGGA	
MYB122_Fw	AT1G74080	ACCTCTTCGAATCTCCCCATC	MYB122
MYB122_Rv		AACTTCATTGATCGGCGTCAC	
SAND_Fw	AT2G28390	AACTCTATGCAGCATTTGATCCACT	SAND
SAND_Rv		TGATTGCATATCTTTATCGCCATC	
TGG1_Fw	AT5G26000	CGGACGTACACACTGCCTTGA	TGG1
TGG1_Rv		TGGACCAGGAGCATGACCAG	
MAM1_Fw	At5g23010	GAGAAATTGAACGCTGTCTTCTCAC	MAM1
MAM1_Rv		AGCCGTTAGACTTTAAACCGTTAGC	
CYP79B3_Fw	At2g22330	CTCCTTCTTCCTTGCAAATGGA	CYP79B3
CYP79B3_Rv		GAGAATCATCAAGAAGCAAAGGG	
CYP83B1_Fw	At4g31500	GGCAACAAACCATGTCGTATCAAG	CYP83B1
CYP83B1_Rv		CGTTGACACTCTTCTTCTCTAACCG	
CYP83A1_Fw	At4g13770	TTCAAGAGGTTGTCAATGAGACGC	CYP83A1
CYP83A1_Rv		CTACAATATCCAAGATGACGGCTTT	
GL2_Fw	AT1G79840	ATGAAGCTCGTCGGCATGAGTGGG	GLABRA2
GL2_RV		TGGATTGCCACTGAGTTGCCTCTG	
LHCBI_Fw	At1g29910	TTC CCT GGA GAC TAC GGA TG	LHCBI
LHCB1_Rv		CCC ACC TGC TTG GAT AAC T	

**Table A2:** **Accurate mass measurements of sodiated molecular ions of ω-methylsulphinylalkyl-nitriles using UPLC/ESI(+)-QTOFMS**.

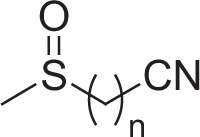

***n***	**Elem. comp**.	**Ret. time [s]**	**Sodiated molecular ion [M+Na]^+^**		
			***m/z* calculated**	***m/z* measured**	**Error [ppm]**
4	C_6_H_11_NOS	68	168.04536	168.0448	+3.5
7	C_9_H_17_NOS	275	210.09231	210.0915	+3.7
8	C_10_H_19_NOS	334	224.10796	224.1084	−1.8

**Table A3:** **Collision-induced dissociation mass spectra [*m/z* (relative intensity)] obtained from protonated molecular ions of ω-methylsulphinylalkylnitriles using UPLC/ESI(+)-QTOFMS**.

***n***	**CE [eV]**	**[M+H]^+^**	**[M+H−CH_4_SO]^+^**	**[M+H−CH_4_SO−NH_3_]^+^**	**Other ions**
4	10	146 (73)	82 (75)	−	129 (42, C_6_H_9_OS^+^), 101 (93, C_5_H_9_S^+^), 98 (77, C_5_H_8_NO^+^), 87 (100, C_4_H_7_S^+^), 75 (11, C_3_H_7_S^+^), 70 (4, C_4_H_8_N^+^), 65 (9, CH_5_SO^+^), 61 (C_2_H_5_S^+^), 55 (15, C_4_H^+^_7_)
7	15	188 (36)	124 (100)	107 (40)	105 (7, C_8_H^+^_9_), 96 (8, C_6_H_10_N^+^), 91 (12, C_7_H^+^_7_), 82 (23, C_5_H_8_N^+^), 81 (18, C_6_H^+^_9_), 79 (54, C_6_H^+^_7_), 77 (7, C_6_H^+^_5_), 65 (3, CH_5_SO^+^)
8	15	202 (57)	138 (100)	121 (23)	110 (7, C_7_H_12_N^+^), 105 (2, C_8_H^+^_9_), 96 (36, C_6_H_10_N^+^), 95 (19, C_7_H^+^_11_), 93 (61, C_7_H^+^_9_), 91 (5, C_7_H^+^_7_), 82 (21, C_5_H_8_N^+^), 81(11, C_6_H^+^_9_), 79 (16, C_6_H^+^_7_), 77 (4, C_6_H^+^_5_), 65 (4, CH_5_SO^+^)

Elemental compositions of observed fragment ions are supported by accurate mass measurements (±10 ppm). CE, collision energy.

## Abbreviations

GS: glucosinolates
IGS: indolic glucosinolates
AGS: aliphatic glucosinolats
ITCs: isothiocyanates.
